# Investigating the level of vitamin D receptor gene expression in two tumoral and healthy breast tissues in breast cancer patients and its association with prognostic factors

**DOI:** 10.1186/s43046-024-00215-5

**Published:** 2024-04-15

**Authors:** Maryam Bahador, Marjan Saeedi Nejad, Shahriar Dabiri, Mohammad Hasan Larizadeh, Maryam Fekri Soofiabadi

**Affiliations:** 1 Department of Radiation Oncology, Kerman University of Medical Sciences, Kerman, Iran; 2 Department of Pathology, School of Medicine, Kerman University of Medical Sciences, Kerman, Iran; 3 Pathology and Stem Cell Research Center, School of Medicine, Kerman University of Medical Sciences, Kerman, Iran

**Keywords:** Vitamin D expression level, Breast cancer, Prognostic indicators

## Abstract

**Background:**

Breast cancer is one of the most common cancers known among women. This study aimed to investigate the level of vitamin D receptor gene expression in two tumoral and healthy breast tissues in breast cancer patients and its association with prognostic factors.

**Methods:**

This descriptive cross-sectional study was conducted in 2022 on 50 patients with high suspicion of breast cancer who were candidates for mastectomy and lumpectomy in a learning hospital. From the patients, two tissue samples were prepared, and there was a total of 100 samples. The samples were subjected to H/E staining and evaluated by a pathologist. The presence or absence of malignancy in each sample was confirmed by two pathologists, and HER2/ER/PR indices were determined. Descriptive and analytical statistical methods and SPSS version 22 software were used.

**Results:**

The average age of the patients was 51.60 ± 11.22 years old, and the average tumor size was 3.17 ± 1.28. Most tumors were grade 2 (48%). The expression of HER2, ER, and PR was positive in 24, 64, and 54%, respectively. The largest number of cases were in stage 2A. The expression level of vitamin D receptor (VDR) gene in healthy tissue (2.08 ± 1.01) was higher than tumoral tissue (0.25 ± 1.38) (*P* = 0.001). In tumoral and healthy tissue, VDR expression was not significant according to tumor grade, HER2, ER, PR, LVI, LN, disease stage, age, and tumor size.

**Conclusions:**

The expression level of VDR in healthy tissue was significantly higher than tumoral tissue. However, there was no significant relationship between VDR and tumor grade, HER2, ER, PR, LVI, LN, disease stage, age, and tumor size.

## Background

Breast cancer is the most common cancer known among women (not including skin cancers), and 1.7 million people in the world are diagnosed with this disease every year. After lung cancer, breast cancer is the second leading cause of cancer-related death in women [1]. Breast cancer is usually divided into two main categories: ductal and lobular carcinomas, in addition to histological structure, based on molecular characteristics, such as the expression of progesterone receptor (PR), estrogen receptor (ER) and human epidermal growth factor receptor (HER-2), breast cancers to luminal-A (ER + ; PR + / − ; HER-2 −), luminal-B (ER + ; PR + / − ; HER-2 +), HER-2 (ER − ; PR − ; HER-2 +), and basal like (ER − ; PR − ; HER-2 −) [2].

The results of some studies show that low levels of vitamin D are related to a number of cancers, especially breast cancer [3]. Therefore, the role of vitamin D in breast cancer has been taken into consideration, and it has been tried to use this factor in determining the prognosis and treatment of breast cancer patients. Subsequent studies have shown that a decrease in the serum level of vitamin D is associated with an increase in the risk of recurrence and mortality in patients with breast cancer [4], and an increase in the serum level of vitamin D is associated with a decrease in mortality from breast cancer [5].

In addition to the classical and well-known form of vitamin D, nonclassical forms of this vitamin and its receptor (VDR) have been identified in other tissues, including breast tissue in recent studies [6]. Genetic studies have shown the role of vitamin D receptor in the differentiation process of normal breast tissue, and it seems that the existence of polymorphisms of VDR can be considered as a risk factor in breast cancer [7]. In more detailed histological investigations, it seems that in the process of breast cancer, vitamin D signaling and its production pathway are disturbed, and cells lose the ability to make the active form of vitamin D, while at the same time the ability to destroy this vitamin increases in cancer cells [8]. Therefore, careful studies of vitamin D and its receptor in healthy and tumoral breast tissue have attracted the attention of researchers.

In Townsend et al. study, natural breast tissue had the ability to produce active vitamin D and was considered a local antiproliferative supplier. On the other hand, a kind of resistance to vitamin D was observed in cancer tissue, and dysregulation in 24-hydroxylase in cancer tissue could cause the conversion of active forms of vitamin D into less active forms [9]. In the study by McCarthy et al., the expression level of hydroxy vitamin D-1-α-hydroxylase-25 in healthy cells adjacent to cancer tissue was decreased compared to normal cells of healthy individuals, and the expression of vitamin D receptor in cancer cells was increased. They also proposed that the detection of αOHase1 reduction in an individual might be a predictor of cancer in that individual [10]. In another study, in tumoral cells, the signaling and regulation of vitamin D synthesis pathway were disturbed, and the ability to synthesize the active form of this vitamin was lost in cancer tissue [8]. Suetani et al. found that normal and noncancerous breast tissue responded similarly to vitamin D administration. While the response of cancer tissue is different and generally does not respond to the inactive precursor form of this vitamin [11]. The results of another study also showed that vitamin D signaling is disturbed in cancer tissue, which makes the anticancer activity of vitamin D ineffective [12].

By reviewing the studies, it seems that the anticancer role of vitamin D and the disruption of the signaling pathway and the activity of this vitamin in the process of breast cancer have been investigated and confirmed by researchers. However, the place of these changes in the clinical interpretation of the patients’ condition, the attended place in the process of early diagnosis, treatment, and finally the follow-up after the treatment are still being discussed and less addressed. Therefore, the aim of the present study was to investigate the level of vitamin D receptor gene expression in two tumoral and healthy breast tissues in breast cancer patients and its association with prognostic factors method.

## Methods

### Study design and participants

This descriptive cross-sectional study was conducted in 2022 on 50 patients with high suspicion of breast cancer who were candidates for mastectomy and lumpectomy in a learning hospital. The sample volume was calculated based on reference number [13].

### Data collection

From each of the 50 patients with breast cancer who were finally included in the study, two tissue samples were prepared (one from the tumoral part of the tissue and the other from the adjacent healthy tissue), which totaled 100 samples. The studied samples were fresh samples of breast cancer along with its healthy (normal) control from the breast tissue itself. Surgical biopsy samples were collected after obtaining informed consent from the patients in Afzalipour Hospital, Kerman, Iran, and then were prepared for H&E (hematoxylin & eosin) and IHC (immunohistochemistry) staining. H&E slides were reviewed by two pathologists double-blindly.

#### IHC staining

Dehydrated, deparaffinized sections along with retrieval buffer were microwaved for 20 min (3 min at 850 W; 17 min at 180 W), and then endogenous peroxidase was blocked for 10 min with 0.5% H202. The sections were incubated for 1 h at room temperature with monoclonal antibodies, in this way: HER2-neu (1:100; DAKO), PR (1:100; DAKO, Clone PgR 636), and ER (1:50; DAKO, Clone 1D5): ready to use. Slides were rinsed with wash buffer for 5 min; this step was repeated twice between all stages. Envision polymer (30 min) was added using 3,3′-diaminobenzidine (DAB) as the chromogen (10 min) after these steps hematoxylin staining for 2 min, dehydration, and mounting the slides. Then, the obtained slides were scored by two pathologists according to the standard scores for ER, PR, and c-erb-B2as defined by WHO.

#### Extraction of RNA from the samples

Biopsy samples were immediately placed in a sterile RNase-free microtube in a nitrogen tank (temperature − 186 °C). After being transferred to the laboratory, the tissue samples were kept at − 80 °C until the RNA extraction stage. To extract RNA, the tissue sample was powdered in a completely sterile mortar containing liquid nitrogen and transferred to a 1.5-µl microtube. One milliliter of TRIzol lysis solution was added to the sample and incubated for 5 min at room temperature. Then 200 µl of chloroform was added to the solution, mixed, and homogenized by screwing the microtube. Then it was centrifuged at 13,000 rpm at 4 °C for 15 min to create three phases. The supernatant phase containing RNA was transferred to another microtube, and 500 µl of isopropanol was added to the solution, shaken back and forth, and centrifuged for 10 min at 13,000 rpm. Then, the supernatant solution was emptied so that the sediment remained at the bottom of the microtube, and after that, 70% ethanol was added to the sediment and centrifuged at 10,000 rpm for 5 min. Then, the supernatant solution was emptied, and the sediment was allowed to dry for 10 min at room temperature. After those 30 µl of water, DNase-RNase free was added to it. After RNA extraction, its quantity and quality were checked by UV spectrum and agarose gel electrophoresis photometric methods.

Before performing the reverse transcription reaction, it was necessary to treat the extracted RNA with DNase 1 enzyme to remove the residual DNA in the medium. For this, 1 µg of RNA, 1 µl of buffer, 1 µl of DNase enzyme, and up to 9 μl of water were added to it and incubated for half an hour at 37°. Then 1 μl of EDTA was added to it and incubated for 10 min at 65 °C.

#### cDNA synthesis

The treated RNA was used as template for reverse transcription reaction. The reverse transcription reaction was performed in by adding 1 μl of oligo primer, 1 μl of random hexamer on RNA with concentration of 1 µg and up to 12 µl of water at 65 °C for 5 min, and then 4 µl of 5 × buffer, 1 μl of RNase inhibitor (RI),1 μl RT enzyme, and 2 μl of dNTPs mixture in a temperature program of 60 min at a temperature of 42 °C in Applied Biosystem StepOne (ABI).

#### Quantitative real-time PCR

After performing the reverse transcription reaction, in order to proliferate the desired fragment, the PCR reaction was performed on the product of the reverse transcription reaction. In this study, with emphasis on VDR gene, primer design was done using vector NIT and oligo program bioinformatics software. In addition, sequences were compared and aligned with BLAST Internet software on NCBI website and GeneBank database. Finally, the primer was designed so that gene expression can be checked by real-time PCR method using Cyber Green method.

- 5′-TGTTGCTGAAATCGCTGACG-3′
- 5′-CATTGTCATCCATTTGCTGCT-3′

To perform the real-time PCR reaction, the following materials were added in a sterile microtube:

- Cyber Green mix, 12.5 μl
- cDNA, 2 μl
- Upstream primer, 0.5 μl
- Downstream primer, 0.5 μl

Sterile deionized water was added to the final volume of 25 μl according to the temperature program of Master Mix Cyber Green protocol.

Then gene expression was checked by real-time PCR method. CT (cycle threshold) sigmoid diagram of each group of biopsy samples (with HER2, PR, ER index) was compared with VDR primer and beta-actin (housekeeping gene).

Vitamin D receptor (VDR) mRNA expression was used to measure tissue vitamin D levels. This expression level of vitamin D receptor was measured by real-time-PCR method in tumoral breast tissue and healthy parts of the same person. Finally, the level of vitamin D in tumoral and healthy tissues, the degree of difference, and their relationship with the prognostic indicators of people were compared, and the results were reported.

Demographic information of the patients, size of the mass, definitive histological diagnosis of the mass, the presence of metastasis to lymphatic and vascular structures, the presence of lymphatic metastasis, and tumor grade were determined and recorded.

### Statistical analysis

Descriptive statistics (frequency, relative frequency, mean, and standard deviation), analytical statistics (independent *t*-test, chi-square, or Fisher), and SPSS software version 22 software used to analyze the data. A significance level of 0.05 was considered.

## Results

**Fig. 1:**
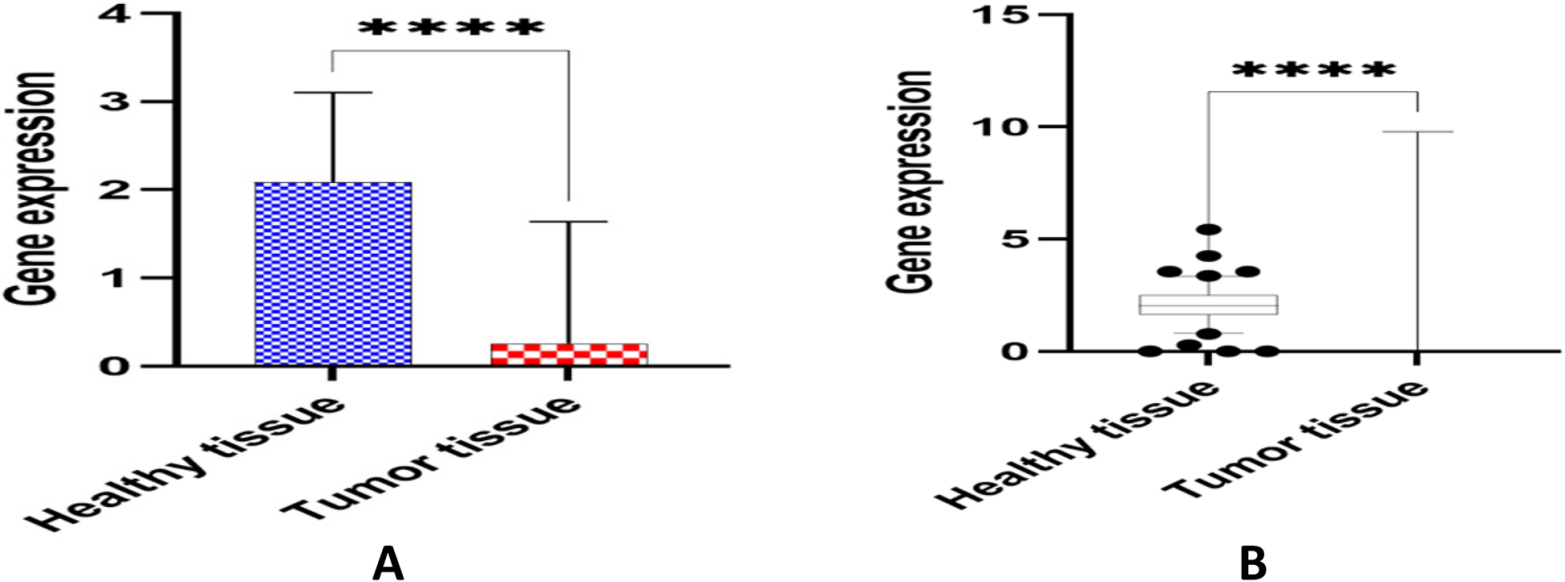
**A** Average and standard deviation. **B** Range and quartiles of VDR gene expression in healthy and tumor tissue

In this study, 50 patients diagnosed with breast cancer were examined. One pair of tissue samples, one from the center of the tumor, and another from the healthy part around the tumor were prepared from the breast tissues of each person. The average age of the patients was 51.60 ± 11.22 years, and the average tumor size was 3.17 ± 1.28. Most of the examined tumors were grade 2 (48%). The expression of HER2, ER, and PR was positive in 24, 64, and 54% of cases, respectively. The highest number of cases was in stage 2A (Table 1). The expression level of vitamin D receptor gene in healthy tissue (2.08 ± 1.01) was higher than tumoral tissue (0.25 ± 1.38). This difference was statistically significant (*P* = 0.001) (Fig. 1).

**Table 1:** Frequency distribution of tumor grade variables, HER2, ER, PR, LVI, LN, and disease stage

**Variable**		**Frequency**	%
**Tumor grade**	**Grade 1**	8	16
	**Grade 2**	24	48
	**Grade 3**	18	36
**HER2**	**Positive**	12	24
	**Negative**	38	76
**ER**	**Positive**	32	64
	**Negative**	18	36
**PR**	**Positive**	27	54
	**Negative**	23	46
**LN**	**N0**	30	60
	**N1**	11	22
	**N2**	8	16
	**N3**	1	2
**LVI**	**Positive**	48	96
	**Negative**	2	4
**Disease Stage**	**1A**	3	6
	**2A**	27	54
	**2B**	7	14
	**3A**	11	22
	**3B**	1	2
	**3C**	1	2

*HER2* Human epidermal growth factor receptor 2, *ER* Estrogen receptor, *PR* Progesterone receptor, *LN* Lymph node, *LVI* Lymphovascular invasion

In tumoral and healthy tissue, the expression level of vitamin D receptor gene was not significant according to tumor grade, HER2, ER, PR, LVI, LN, and disease stage (Table 2).

**Table 2:** Examination of vitamin D receptor gene expression levels in two tumoral and healthy tissues according to tumor grade, HER2, ER, PR, LVI, LN, and disease stage

Tissue	Variable		Vitamin D receptor gene expression level				
			**Average**	**Standard deviation**	**Mean**	**Quartile range**	***P***
**Tumoral**	**Tumor grade**	**1**	1.23	3.45	0.01	0.02	0.9
		**2**	0.04	0.08	0.01	0.04	
		**3**	0.1	0.20	0.01	0.08	
**Healthy**		**1**	2.35	0.7	2.08	1.21	0.58
		**2**	2.04	0.79	2.09	0.98	
		**3**	2	1.37	1.98	1.35	
**Tumoral**	**HER2**	**Positive**	0.04	0.08	0.01	0.03	0.73
		**Negative**	0.32	1.58	0.01	0.05	
**Healthy**		**Positive**	2.32	1.02	2.25	0.92	0.19
		**Negative**	2	1.01	2.02	0.9	
**Tumoral**	**ER**	**Positive**	0.36	1.72	0.01	0.04	0.66
		**Negative**	0.06	0.11	0.01	0.05	
**Healthy**		**Positive**	2.03	0.87	2.05	0.63	0.91
		**Negative**	2.15	1.25	2.04	1.29	
**Tumoral**	**PR**	**Positive**	0.43	1.87	0.01	0.05	0.77
		**Negative**	0.05	0.1	0.01	0.03	
**Healthy**		**Positive**	2.02	0.92	2.04	0.65	0.65
		**Negative**	2.14	1.13	2.05	1.2	
**Tumoral**	**LVI**	**Positive**	0.26	1.41	0.01	0.04	0.52
		**Negative**	0.1	0.19	0.14	0.004	
**Healthy**		**Positive**	2.07	1.03	2.04	0.9	0.79
		**Negative**	2.14	0.31	2.14	1.91	
**Tumoral**	**LN**	**N0**	0.04	0.07	0.01	0.03	0.81
		**N1**	0.94	2.93	0.01	0.05	
		**N2**	0.14	0.27	0.01	0.22	
		**N3**	0.03		0.03	0	
**Healthy**		**N0**	1.96	0.98	2.04	0.9	0.36
		**N1**	2.56	1.11	2.05	1.27	
		**N2**	1.76	0.93	1.98	1.2	
		**N3**	2.78		2.78	0	
**Tumoral**	**Disease stage**	**1A**	0.006	0.004	0.003	0.003	0.66
		**2A**	0.04	0.08	0.01	0.01	
		**2B**	1.47	3.66	0.055	0.42	
		**3A**	0.1	0.23	0.01	0.04	
		**3B**	0.003		0.003	0	
		**3C**	0.03		0.03	0	
**Healthy**		**1A**	2.63	0.86	2.49	1.84	0.35
		**2A**	1.95	1.01	2.05	0.91	
		**2B**	2.52	1.37	2.11	1.35	
		**3A**	2.09	0.6	2.04	0.67	
		**3C**	2.78		2.78	0	

In tumoral and healthy tissue, no significant difference was observed between the age and tumor size and the vitamin D receptor gene expression level (Table 3).

**Table 3:** Examining the expression level of vitamin D receptor gene in two tumoral and healthy tissues according to age and tumor size

Tissue	Variable	Vitamin D receptor gene expression level	
		***r***	***P***
**Tumoral**	**Age**	− 0.12	0.38
**Healthy**	**Tumor size**	− 0.02	0.85
	**Age**	− 0.12	0.39
	**Tumor size**	− 0.13	0.34

## Discussion

In this study, the expression level of vitamin D in tumoral and healthy breast tissue in people with breast cancer was investigated, and its relationship with prognostic indicators was identified. With 2.3 million diagnoses per year, breast cancer is the most commonly diagnosed cancer in women and the leading cause of cancer-related deaths worldwide [14].

Research results show that vitamin D regulates a wide range of biological activities, which are independent of each other, including bone metabolism and cell cycle regulation [15]. Vitamin D exerts its anticancer effects through vitamin D receptor and transcription of target genes such as BRCA1 and P53 [7]. Therefore, investigating vitamin D as a potential factor in reducing the incidence and treatment of breast cancer seems necessary.

In the present study, the average age of the patients was 51.60 ± 11.22 years, and the average tumor size was 3.17 ± 1.28. Most of the examined tumors were grade 2 (48%). The expression of HER2, ER, and PR was positive in 24, 64, and 54% of cases, respectively. The largest number of cases was in stage 2A. In Nemati et al. study, the average age of women with breast cancer was 44.07 ± 7.99 years [16].

The main function of 1,25(OH)2D3 is to maintain calcium and phosphate homeostasis in the body. However, VDR is not only expressed in the intestine, kidney, and bone tissue but also in many other tissues, including cancer. Both in vitro and in vivo clinical studies have shown that 1,25(OH)2D3 modulates various signaling pathways involved in cell proliferation, apoptosis, differentiation, inflammation, invasion and angiogenesis [17].

VDR is expressed in various types of mammary gland cells, including lobular and ductal epithelial cells, where it plays an important role in mammary gland development during puberty, lactation, and pregnancy and periods of maximal tissue growth and remodeling [18]. During puberty in mice, VDR expression was highest in differentiated cells in terminal buds, whereas expression was low in proliferative regions of the mammary gland [19]. In human breast cancer tissue, VDR expression has been reported to be inversely correlated with breast cancer invasiveness. In benign breast lesions, VDR was significantly more expressed than in breast carcinoma lesions (in situ and invasive) [20]. In addition, various groups investigated whether VDR expression could be used as a potential biomarker for cancer progression and survival [18, 21–24]. Recently, higher expression of VDR in breast cancer lesions (both in nucleus and cytoplasm) has been associated with tumor characteristics such as lower grade, smaller size, ER/PR positivity, lower Ki67 expression, and lower risk of breast cancer mortality [18, 21]. In the present study, the level of vitamin D receptor gene expression in healthy tissue was significantly higher than tumoral tissue. However, there was no significant relationship with tumor grade, HER2, ER, PR, LVI, LN, disease stage, age, and tumor size. In Nemati et al.’s study, there was no significant relationship between vitamin D serum level and HER2 receptor. However, the relationship between serum levels of vitamin D and estrogen and progesterone receptors was significant [16].

## Conclusion

According to the present study, the level of vitamin D receptor gene expression in healthy tissue was significantly higher than tumoral tissue. However, there was no significant relationship between VDR and tumor grade, HER2, ER, PR, LVI, LN, disease stage, age, and tumor size. It is recommended to consider a larger sample size in studies. In addition to the tissue level, the serum level of vitamin D should be measured. In addition, a more heterogeneous group should be selected from different sample sizes with higher stages and grades. In line with the design of our study, it is suggested that another study simultaneously measures the serum level of vitamin D and the tumoral tissue. Therefore, it may be possible to understand the mechanism of vitamin D in the tumoral tissue.

## Data Availability

All data generated or analyzed during this study are included in this published article.

## Abbreviations

VDR: Vitamin D receptor
PR: Progesterone receptor
ER: Estrogen receptor
HER-2: Human epidermal growth factor receptor
H&E: Hematoxylin & eosin
IHC: Immunohistochemistry
DAB: Diaminobenzidine
CT: Cycle threshold
LN: Lymph node
LVI: Lymphovascular invasion
